# A metagenome-wide association study of the gut microbiota in recurrent aphthous ulcer and regulation by thalidomide

**DOI:** 10.3389/fimmu.2022.1018567

**Published:** 2022-10-19

**Authors:** Xiang Wang, Kexu Xiong, Fan Huang, Jinqun Huang, Qin Liu, Ning Duan, Huanhuan Ruan, Hongliu Jiang, Yanan Zhu, Lin Lin, Yuefeng Song, Maomao Zhao, Lichun Zheng, Pei Ye, Yajie Qian, Qingang Hu, Fuhua Yan, Wenmei Wang

**Affiliations:** ^1^ Nanjing Stomatological Hospital, Medical School of Nanjing University, Nanjing, China; ^2^ College of Life Sciences, University of Chinese Academy of Sciences, Beijing, China; ^3^ Beijing Genomics Institute (BGI)-genomics, BGI-Shenzhen, Shenzhen, China

**Keywords:** gut microbiota, recurrent aphthous ulcer, metagenome, immunity, thalidomide

## Abstract

Recurrent aphthous ulcer (RAU), one of the most common diseases in humans, has an unknown etiology and is difficult to treat. Thalidomide is an important immunomodulatory and antitumor drug and its effects on the gut microbiota still remain unclear. We conducted a metagenomic sequencing study of fecal samples from a cohort of individuals with RAU, performed biochemical assays of cytokines, immunoglobulins and antimicrobial peptides in serum and saliva, and investigated the regulation effects of thalidomide administration and withdrawal. Meanwhile we constructed the corresponding prediction models. Our metagenome-wide association results indicated that gut dysbacteriosis, microbial dysfunction and immune imbalance occurred in RAU patients. Thalidomide regulated gut dysbacteriosis in a species-specific manner and had different sustainable effects on various probiotics and pathogens. A previously unknown association between gut microbiota alterations and RAU was found, and the specific roles of thalidomide in modulating the gut microbiota and immunity were determined, suggesting that RAU may be affected by targeting gut dysbacteriosis and modifying immune imbalance. In-depth insights into sophisticated networks consisting of the gut microbiota and host cells may lead to the development of emerging treatments, including prebiotics, probiotics, synbiotics, and postbiotics.

## Introduction

Recurring aphthous ulcer (RAU), also known as recurrent oral ulcer, is a condition that affects 20% of the world’s population and is characterized by painful, yellow ulcers in the oral mucosa (1). Frequent or almost continuous recurrence causes terrible difficulties in eating, drinking, swallowing, and speaking, and as a result, it negatively affects the quality of life of RAU patients. Several factors have been proposed as possible causative agents for RAU, including microbial and immune factors, but a definitive etiology of RAU has yet to be clearly established (2).

Characterization of the gut microbiota has become an important research area for human diseases. The important role of dysbacteriosis in a variety of diseases [colorectal cancer, type 2 diabetes, and Behcet’s disease (BD)] has been widely recognized (3–5). The gut microbiota, microbial function, and immune factors, which are generally in a state of dynamic balance, contribute substantially to human health. A previous study indicated that RAU is related to changes in the oral microflora (6), but the relationship between the gut microbiota and RAU is still rarely reported. Moreover, a correlation network study based on metagenomic analysis of the gut microbiota along with immune factor assays of serum and saliva has not been conducted.

Thalidomide has long been used for the treatment of RAU, and can effectively reduce the frequency, number, and pain of ulcers (7). Thalidomide has immunomodulatory, anti-inflammatory and antiangiogenic effects and has been widely applied in the treatment of immune system diseases and malignant tumors (8). Thalidomide is effective in RAU treatment, but the specific mechanism is still unclear. To the best of our knowledge, thalidomide has not been linked to microbiota modulation in the literature.

Pioneering studies on the oral microbiota characteristics of RAU patients were based on 16S rRNA gene amplicon sequencing (9, 10). So far, no individual pathogens have been conclusively shown to be correlative agents of RAU (6). A recent study suggested that RAU occurrence is significantly associated with an increase in *Escherichia coli* and a decrease in *Alloprevotella* abundances (11). However, few metagenomic studies on RAU have been reported. Metagenomic sequencing can provide better genome coverage and obtain genetic diversity, molecular ecological, and microbial function information (12).

In this study, we first investigated the metagenome-wide association of thegut microbiota in RAU and found previously unknown aberrant profiles ofthe intestinal microbiota in RAU and the specific regulatory effects of thalidomide on the intestinal microbiota and immune factors. Our study might improve the understanding of RAU pathogenesis and the possible mechanism of thalidomide in treating the disease, providing novel ideas for precision therapy by supplementing with probiotics, prebiotics, synbiotics and postbiotics.

## Results

### Diversity analysis of the gut microbiota in RAU

**Figure 1:**
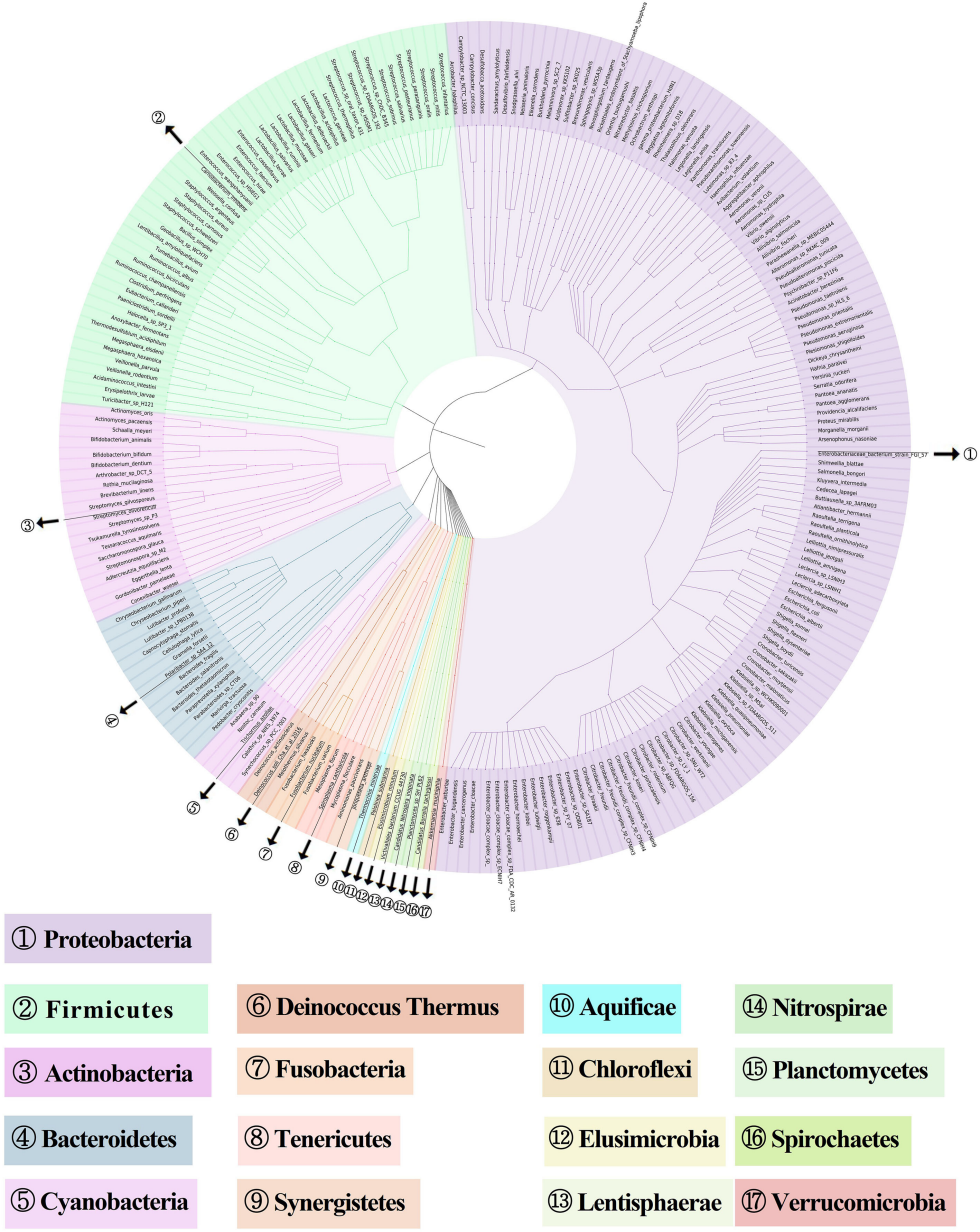
Metagenome-based diversity analysis of RAU gut microbiota. In total, 238 abundant species between RAU patients and healthy controls are shown in a phylogenetic tree according to the color code. The phylums of bacterias are given in the outer circles with circled numbers which including 17 phylums.

**Figure 2:**
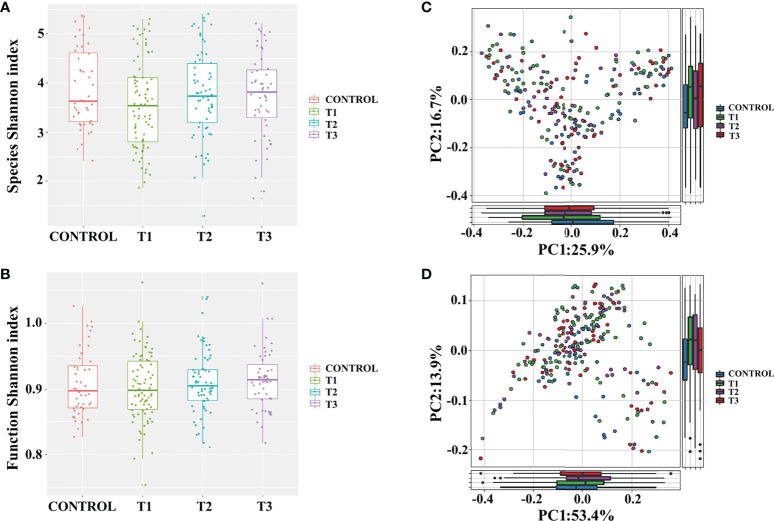
Gut microbiome-associated taxonomic and functional characteristics of RAU patients and healthy controls. **(A)** Taxonomic alpha-diversity of RAU and healthy controls (*P*>0.05). **(B)** Functional alpha-diversity of RAU and healthy controls (*P*>0.05). **(C)** Taxonomic beta-diversity of RAU and healthy controls (*P*>0.05). **(D)** Functional beta-diversity of RAU and healthy controls (*P*>0.05).

To examine the gut microbiota of patients with RAU, we first analyzed fecal samples from 81 patients with refractory RAU [at the visit time (T)1] and 44 age- and sex-matched healthy controls (Control) by metagenomic sequencing. The bacterial diversity of the fecal microbiota in the RAU and control groups is shown in **Figure 1**, and the baseline information of the two groups is shown in **Table S1**. In total, 238 species belonging to 17 phyla (Actinobacteria, Bacteroidetes, Fibrobacteres, Firmicutes, Fusobacteria, Gemmatimonadetes, Proteobacteria, etc.) were detected by comparisons [Control vs. T1, T1 vs. T2, T2 vs. T3, false discovery rate (FDR)≤0.05] (**Table S2**) and there were no differences of gut microbiome-associated taxonomic and functional diversity in the RAU patients and controls (**Figure 2**).

### Taxonomical signatures of the gut microbiota in RAU and regulation by thalidomide

**Figure 3:**
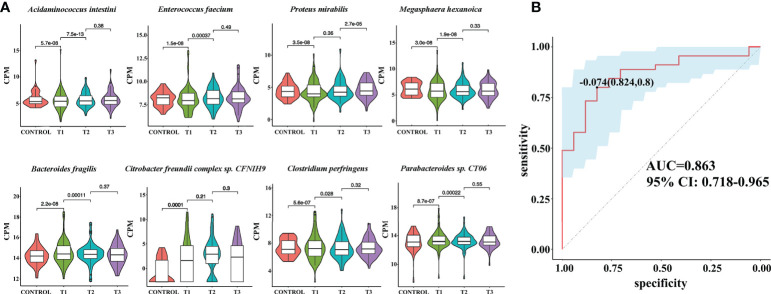
Metagenomic sequencing-based exploration of RAU-associated fecal microbiomes with those of healthy individuals and the prediction model based on fecal microbial species of RAU and healthy controls. **(A)** Violin plot analysis comparing the levels of fecal microbial species in control, T1, T2 and T3 groups (*P*<0.05). The vertical position of each histogram represents the relative expression level of fecal microbial species. **(B)** Receiver operating characteristic curves for fecal microbial species comprising samples from RAU and healthy controls. “0.824” is the sensitivity of the optimal threshold point, and “0.8” is the specificity. “-0.074” is the socre of adaboost modle at the optimal threshold point. The blue area 95% confidence represents the confidence interval.

To reveal the differences in gut microbes between the RAU patients and controls, we used Kraken2 to annotate the sequenced reads and Bracken to correct the species abundance and screened out the species with significant differences between different stages (FDR ≤ 0.005). The results showed that a total of 86 species were significantly differentially abundant between the RAU patients and controls (comparison of Control vs. T1) (FDR ≤ 0.005). A great number of probiotics were significantly depleted, while an array of pathogens were remarkably enriched in the gut microbiota of patients with RAU. *Acidaminococcus intestini*, *Raoultella terrigena*, *Enterococcus faecium*, *Hafnia paralvei* and other probiotics were significantly depleted in the RAU patients (**Figures 3A**, **S1** **)**. *Bacteroides fragilis*, *Parabacteroides* sp. *CT06*, *Enterococcus phage IMEEFm1*, *Enterobacter bugandensis* and other pathogens were significantly enriched in RAU patients (**Figures 3A**, **S1**). Notably, *A*. *intestini*, *Enterococcus faecium*, *Proteus mirabilis* and other probiotics increased in T2 and continued to increase in T3 **Figure 3A**), indicating that thalidomide has an upregulatory effect on the decreased abundances of probiotics and that this effect could be maintained *via* a long-term regulation. Our results showed that *A. intestine* abundance decreased in RAU patients, indicating that *A. intestini* is a potential probiotic. *A. intestini* is knownto be a normal commensal of the human gut (13). Its metabolic end products are acetic acid, butyric acid and propionic acid. The antibiotic-resistant *Enterococcus phage IMEEFm1* has shown highly effective lytic activity against *Enterococcus faecium* (14).

Similarly, one month after taking thalidomide, *B. fragilis*, *E. coli*, *Parabacteroides* sp. *CT06*, *Enterococcus phage IMEEFm1*, and *Citrobacter freundii* abundances were significantly reduced and dropped continuously one month after thalidomide withdrawal (**Figures 3A**, **S1** **)**. These results indicated that thalidomide had a downregulatory effect on the increase in pathogenic bacteria abundance and that this effect could be maintained *via* a long-term regulation. *B. fragilis* is an opportunistic pathogen involved in causing disease in humans under certain conditions, such as disruption of the colon mucosal surface induced by inflammation, trauma, or surgery, and the spread of *B. fragilis* to the bloodstream or surrounding tissues results in clinically significant infection (15).

A previous study also suggested that RAU occurrence is significantly associated with an increase in *E.* coli abundance (11). In this study, thalidomide increased the abundance of potential probiotics, which was maintained after drug discontinuation, while it reduced the abundance of potential pathogenic bacteria, which was maintained after drug discontinuation. Our results suggest that thalidomide can regulate the disturbance of the gut flora, which further suggests that this may be a novel mechanism of thalidomide in the treatment of RAU.

The symbiosis factor of *B. fragilis*, PSA, can directly induce the anti-inflammatory function of regulatory T cells (Tregs) and restrain intestinal T helper 17 (Th17) cell development and responses during commensal colonization (16). Moreover, *B. fragilis* can produce propionic acid to increase Treg cell numbers while decreasing Th17 cell numbers (17, 18). Additionally, *Parabacteroides* produces acetate to alleviate inflammation by reducing neutrophil infiltration (17).

**Figure 4:**
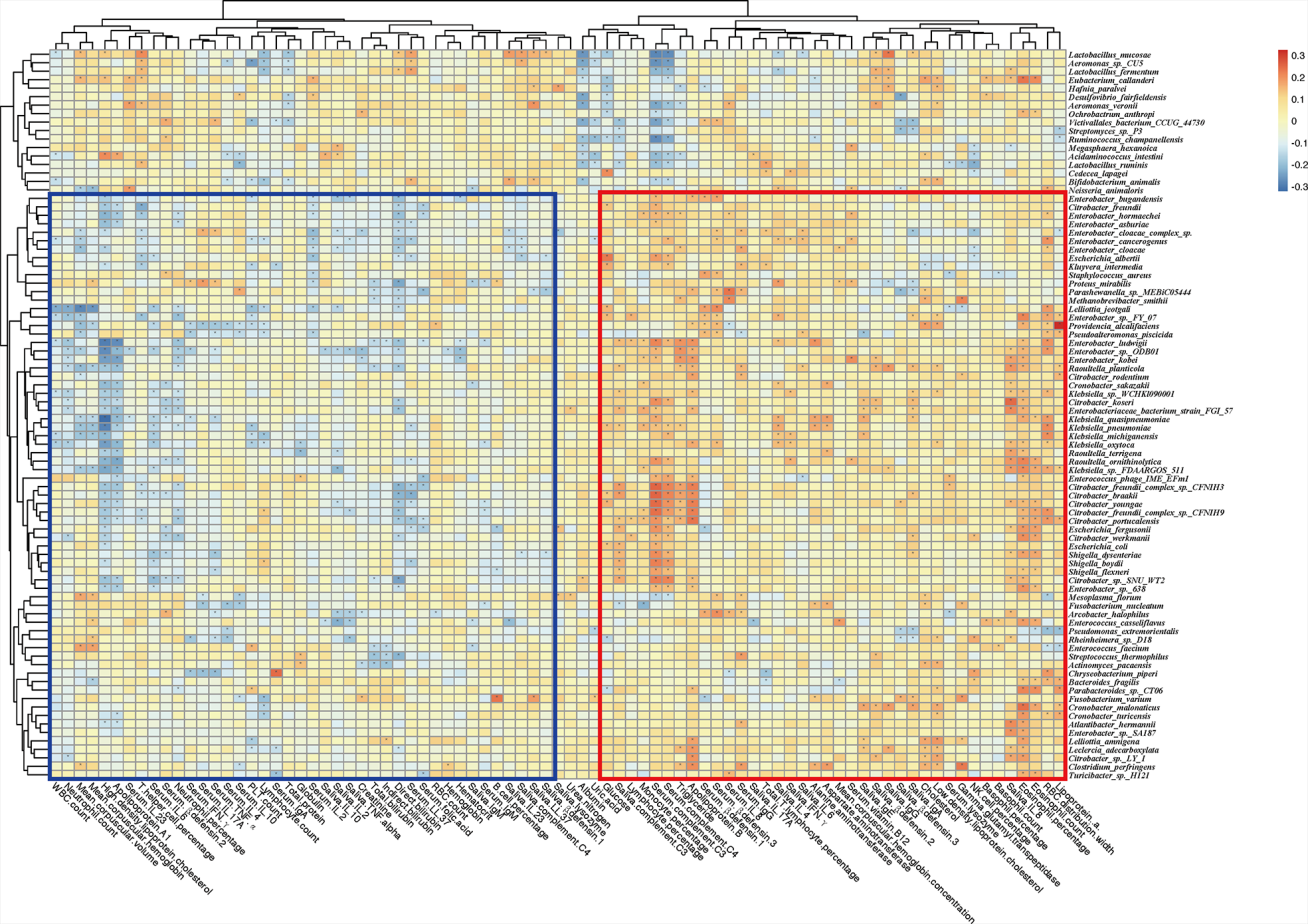
Correlation heatmap of gut microbiome-associated species and the blood and saliva factors between RAU patients and healthy controls. Blue boxes denote the cluster of negative correlation, those encompassed by the red box represent the cluster of positive correlation. The colors are proportional to the correlation strength, the variation from red to blue represent positive to negative trend. “*” means *P*<0.05.

To reveal the key microbial groups leading to gut microbiota variation in RAU, edgeR was used to evaluate the differences between Control and T1 samples (FDR ≤ 0.05). A total of 190 bacterial species were screened out. The area under curve (AUC) value (R function roc) was calculated for each of the 190 species, and 38 species with AUC>0.7 were screened out. Through the ensemble learning method, these bacteria were modeled (adabag package), and the prediction model with an AUC of 0.863 was obtained by 100 iterations (**Figure 3B**). AUC is used to judge the advantages and disadvantages of the prediction model. The closer the model is to one, the more accurate the prediction is. In addition the correlation of gut microbiome-associated species and blood and saliva factors is shown in the heatmap and some distinct modules could be found (**Figure 4**).

### Functional signatures of the gut microbiota in RAU

**Figure 5:**
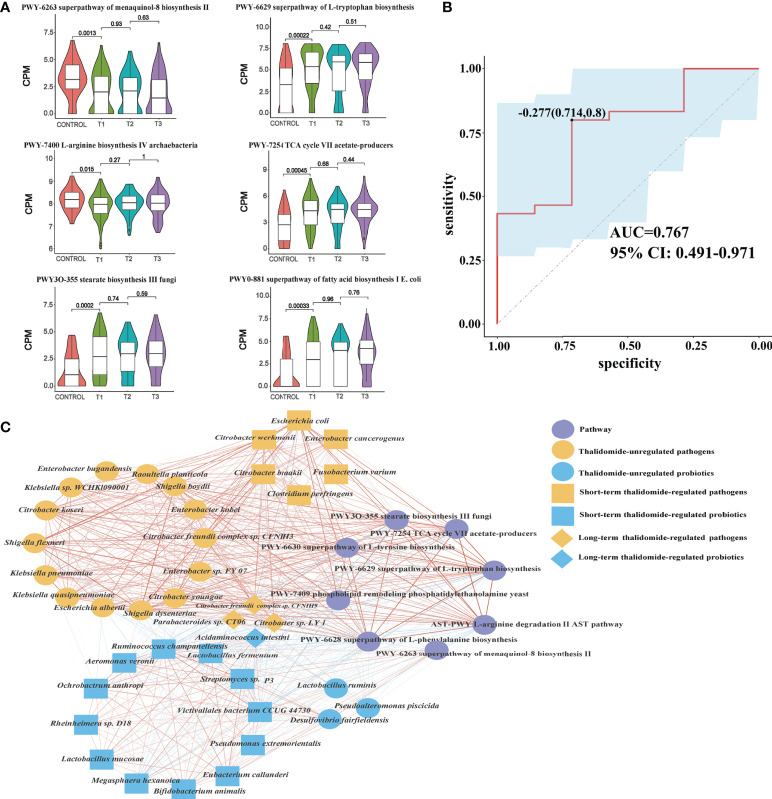
Contributional diversity of fecal microbiome pathways. **(A)** Violin plot analysis comparing the levels of fecal pathways in control, T1, T2 and T3 groups (*P*<0.05 between control and T1). **(B)** Receiver operating characteristic curves for fecal microbial pathways comprising samples from RAU and healthy controls. “0.714” is the sensitivity of the optimal threshold point, and “0.8” is the specificity. “-0.277” is the socre of adaboost modle at the optimal threshold point. The blue area 95% confidence represents the confidence interval. **(C)** Abundance-based species-pathways correlation network enriched in RAU patients and healthy individuals. Two nodes are linked if they are related. The edge width is proportional to the correlation strength. Nodes with the different color and shapes are classified in the different effective order level with Thalidomide.

We used HumanN3 to generate a functional map of the gut microbiota, and our results indicated that there were 90 pathways with significantly distinct abundance between RAU patients and healthy individuals (FDR ≤ 0.05). Among these pathways, menaquinol-8_biosynthesis_II, L−arginine_biosynthesis_IV_archaebacteria, folate_transformations_II, chorismate_biosynthesis_from_3−dehydroquinateand other pathways were significantly depleted in RAU patients at T1 compared with those in the controls (**Figures 5A**, **S2**). Acetylene degradation, TCA cycle VII acetate production, phytate degradation I and other pathways were significantly enriched at T1 compared with those in the controls and positively correlated with pathogenic bacteria (**Figures 5A**, **S3**). L-arginine mediates an important function, maintaining intestinal barrier function and inflammation-associated immunosuppression. Pathways related to short-chain fatty acid (SCFA) and L-arginine synthesis play a significant role in shaping the gut microbiota and innate immunity, thus improving gut development and protecting against pathogenic infection (19). Dietary L-arginine supplementation alleviates liver injury caused by *E. coli* LPS (20), activates intestinal innate immunity (21), and protects against deoxynivalenol-induced toxicity (22).

In particular, there was a positive correlation between probiotics and menaquinol-8_biosynthesis_II, which were significantly depleted at T1 compared with those in the controls. We found that this pathway was negatively correlated with some pathogenic bacteria, such as *E. coli*. It has been reported that cytochrome bo(3) [cyt bo(3)] is one of the three terminal oxygen reductases in the aerobic respiratory chain of *E. coli* and maintains the activity of ubiquitin oxidase through the menaquinol-8 pathway. A potential explanation is that the decrease in the abundance of the menaquinol-8 pathway could induce cyt bo(3) dysfunction or interruption or decrease the function of the aerobic respiratory chain of *E. coli* and enhance micro-oxygen or anaerobic conditions, initiating *E. coli* pathogenicity and RAU (23).

The tricarboxylic acid cycle (TCA) cycle (24) has long been considered a “housekeeping” pathway in *E. coli* and *Salmonella enterica*, and the pathway is highly regulated at the transcriptional level and responds to respiratory conditions. Glyoxylate bypass has long been known to be essential for growth on carbon sources such as acetate or fatty acids. Strains lacking this pathway fail to grow on these carbon sources, since acetate carbon entering the TCA cycle is quantitatively lost as CO_2,_ resulting in the lack of a means to replenish the dicarboxylic acids consumed in amino acid biosynthesis. A microbial production platform has been developed in *E. coli* to synthesize D-glyceric acid from D-galacturonate (24). The use of adequate probiotic lactobacilli, i.e., homolactic and/or facultatively heterolactic l-lactic acid-producing lactobacilli (25), reduces the amounts of intestinal bacteria, toxic metabolites, D-lactic acid and ethanol by fermentative production of the nontoxic l-lactic acid from glucose.

Accordingly, through functional analysis, we found that the beneficial bacteria were negatively correlated with the L-tryptophan biosynthesis pathway, while pathogenic bacteria were positively correlated with the L-tryptophan biosynthesis pathway. A previous study (26) confirmed that some *E. coli* variants can promote an increase in indole and tryptophan production. Tryptophan and 5-hydroxyindomeacetic acid have been found to be significantly enriched in patients with colorectal cancer, indicating that the tryptophan metabolic pathway is was closely related to anti-inflammatory effects (27). Changes in the microbiome regulate the host immune system by regulating tryptophan metabolism. In addition, tryptophan has profound effects on gut microbiome composition, microbial metabolism, the host immune system, host-microbiome interplay, and hostimmune system-gut microbiome interactions. Our results indicated that both *E. coli* and the tryptophan pathway were significantly enriched in RAU, suggesting that changes in the tryptophan metabolic pathway along with *E. coli* abundance were closely related to the occurrence of RAU.

**Figure 6:**
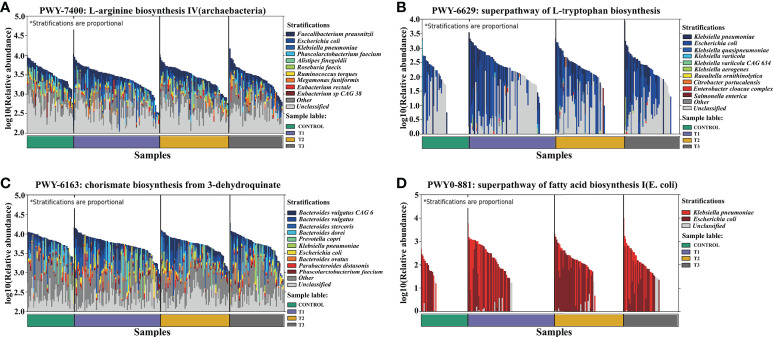
Dominant species contributing to metabolic pathways. **(A)** L−arginine biosynthesis IV (archaebacterial). **(B)** Superpathway of L-tryptophan biosynthesis. **(C)** Chorismate biosynthesis from 3−dehydroquinate. **(D)** Superpathway of fatty acid biosynthesis I (*E. coli*).

**Figure 7:**
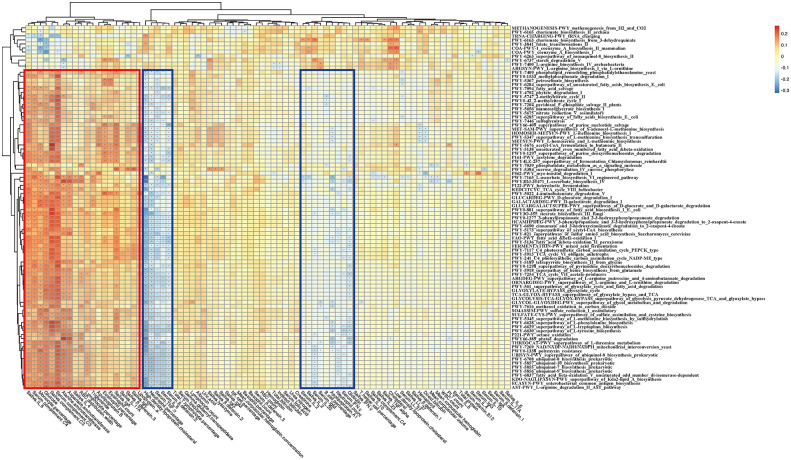
Correlation heatmap of gut microbiome-associated metabolic pathways and the blood and saliva factors between the RAU patients and health controls. Blue boxes denote the cluster of negative correlation, those encompassed by the red box represent the cluster of positive correlation. The colors are proportional to the correlation strength, the variation from red to blue represent positive to negative trend. “*” means *P*<0.05.

To reveal the key pathways leading to functional variation of the gut microbiota in RAU, differences in Control and T1 samples were evaluated by limma (FDR ≤ 0.05), and a total of 90 pathways were screened out. The AUC value (R function roc) was calculated for each of the 90 pathways, and 5 pathways (AUC>0.7) were selected. Through the ensemble learning method, these pathways were modeled (adabag package) and iterated 100 times to obtain the prediction model with an AUC of 0.767, including pathways (**Figure 5B**). The correlation network of relevant pathways and species between the RAU patients and health controls is shown in **Figure 5C**. The main contributional pathways of feces microbiota are shown in **Figure 6** and the correlation heatmap of gut microbiome-associated metabolic pathways and blood and saliva factors is shown in **Figure 7**.

### Regulatory effects of thalidomide on immune factor levels in RAU

**Figure 8:**
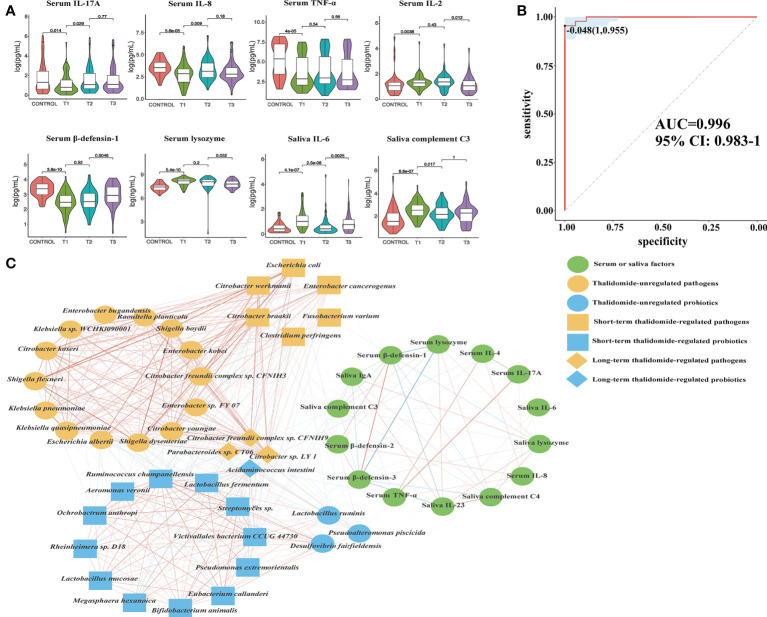
Contributional diversity of fecal microbiome cytokines. **(A)** Violin plot analysis comparing the levels of serous and salivary cytokines in control, T1, T2 and T3 groups (*P*<0.05 between control and T1). **(B)** Receiver operating characteristic curves for fecal microbialcytokines comprising samples from RAU and healthy controls. “1” is the sensitivity of the optimal threshold point, and “0.955” is the specificity. “-0.048” is the socre of adaboost modle at the optimal threshold point. The blue area 95% confidence represents the confidence interval. **(C)** Abundance-based species-cytokines correlation network enriched in RAU patients and healthy individuals. Two nodes are linked if they are related. The edge width is proportional to the correlation strength. Nodes with the different color and shapes are classified in the different effective order level with Thalidomide.

To measure the alterations in serum and saliva levels of cytokines, antimicrobial peptides, and immunoglobins between groups, ELISAs were used. The results showed that the levels of interleukin (IL)-17A, tumor necrosis factor (TNF)-α, IL-2, IL-4, IL-8, β-defensin-2, β-defensin-3, immunoglobulin (Ig)A, and complement C3, C4 in serum were significantly different between comparisons (**Figures 8A**, **S4**). The levels of IL-6, IL-23, interferon (IFN)-γ, lysozyme, IgA, IgG, IgM, and complement C3, C4 in saliva were significantly different between groups (**Figures 8A**, **S5**). Interestingly, we detected that TNF-α, IL-4, IL-8, IL-17A, β-defensin-1, β-defensin-2, and β-defensin-3 levels in serum were positively correlated with probiotic abundances. However, lysozyme in serum and lysozyme, IFN-γ, IL-6, IL-23, IgA, complement C3, and C4 in saliva were positively correlated with pathogenic bacteria. Most notably, serum IL-17A levels significantly decreased at T1 compared with those in the controls, suggesting that systemic and protective Th17 inflammation was inhibited in the RAU. One month after taking thalidomide, serum IL-17A levels significantly increased at T2 compared with T1 (**Figures 8A**), indicating that thalidomide can rescue protective Th17 inflammation and immunity against pathogens. Moreover, our results indicated that serum IL-4 and IL-8 levels significantly increased at T2 compared with T1 (**Figures 8A**, **S4**). Additionally, our results indicated that serum IL-23 level and saliva IL-6, IFN-γ, lysozyme, complement C3, and C4levels significantly decreased at T2 compared with T1 (**Figures 8A**, **S4**, **S5**). Since most inflammatory and autoimmune diseases involve Th17 generation, it could be proposed that one of the novel mechanisms of action of thalidomide and its analogs could be blocking this cytokine, enhancing an anti-inflammatory response (28).

Digestive tract dysbacteriosis may cause diseases. Most recently, studies have demonstrated that finely tuned crosstalk between the microbiota, immune cells, and the epithelium is critical for the maintenance of the mucosal architecture and homeostasis (29–31). An increasing body of evidence suggests that perturbations of the mucosal microbiota can modulate innate and adaptive immune responses, with inflammation arising due to a reduction in the number of symbiont microorganisms and/or an increase in the number of pathobiont microorganisms (commensal bacteria with pathogenic potential) (32, 33). For example, one mechanism by which these microbes regulate immunity is by controlling Tregs and Th17 cells (34). In addition, the epithelium recognizes and responds to the microbiota, and in turn, microbial dysbiosis and associated metabolite alterations destroy the integrity of the mucosal epithelium and its barrier functions (35). The protective effects of the newly identified lineage of Th17 cells against pathogens such as *E. coli*, *Klebsiella pneumoniae*, *Citrobacter rodentium* and *Candida albicans* indicate the capacity of Th17 cells to confer protection against extracellular bacterial and fungal pathogens.

The immunopathogenesis of RAU probably involves a cell-mediated immune response mechanism including TNF-α (36). TNF-α, a major inflammatory mediator, induces regulation of immune cells and initiation of the inflammatory process to protect the host from pathogens. Our data suggest that serum TNF-α levels were negatively correlated with the tryptophan, tyrosine and phenylalanine biosynthesis pathways. TNF-α is strongly modulated by microbial metabolism and degradation of tryptophan to tryptophol (37). Changes in the microbiota stimulate the immune system of the host by regulating tryptophan and other amino acid metabolism, which may be accompanied by changes in factors such as TNF-α and IL-17. The use of thalidomide plays a certain role in the regulation of the gut microbiota and immune system, contributing to the recovery of homeostasis in the host.

To reveal the key immune factors leading to functional variation of the gut microbiota in RAU, differences in Control and T1 samples were evaluated by limma (FDR ≤ 0.05), and these immune factors were modeled (adabag package) and iterated 100 times to obtain the prediction model with an AUC of 0.996 (**Figure 8B**). All metagenomic results in this study are summarized in **Table S1**. The correlation network of relevant factors and species is shown in **Figure 8C**. Given that the entire community of microbial inhabitants in the digestive tract influences immune response balance and epithelial barrier function, our results suggested that RAU could potentially be the outcome of microbiota dysbiosis due to homeostatic disturbance of host-microbe interactions.

### Relapsable characterization of gut microbiota after thalidomide withdrawal

**Figure 9:**
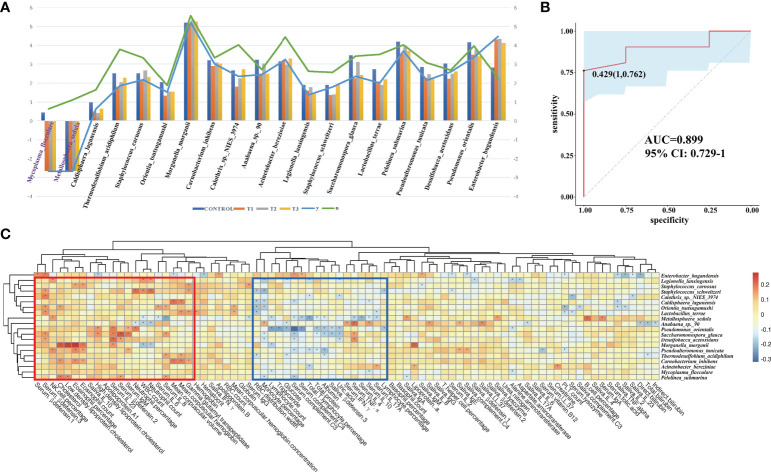
Relapsable characteristics of gut microbiota in RAU patients after thalidomide withdrawal. **(A)** The significantly different species between the replasable and replase-free individuals. **(B)** Receiver operating characteristic curves for fecal microbial species comprising samples from replasable and replase-free individuals. “1” is the sensitivity of the optimal threshold point, and “0.762” is the specificity. “0.429” is the socre of adaboost modle at the optimal threshold point. The blue area 95% confidence represents the confidence interval. **(C)** Correlation heatmap of relapse-associated species and the blood and saliva factors between replasable patients and replase-free individuals. Blue boxes denote the cluster of negative correlation, those encompassed by the red box represent the cluster of positive correlation. The colors are proportional to the correlation strength, the variation from red to blue represent positive to negative trend. “*” means *P*<0.05.

At T3 the taxonomic differences between relapsable and relapse-free individuals were calculated by edgeR (FDR<=0.05), and a total of 20 bacteria were screened out as shown in **Figure 9A**. According to the results, the reduction of *Mycoplasma flocculare* or *Metallosphaera sedula* is more likely to cause relapse, suggesting that these two bacteria may be probiotics to prevent relapse. The AUC value (R function roc) was calculated for each of the species, and 10 species with AUC>0.7 were screened out. The prediction model with an AUC of 0.899 was obtained by 100 iterations (**Figure 9B**), meaning that these bacteria have about 89.9% accuracy of predicting recurrence. The correlation heatmap of relapse-associated species and blood and saliva factors between replasable patients and replase-free individuals is shown in **Figure 9C**.

## Discussion

Evidence that the gut microbiota contributes to the development of RAU is accumulating. Thus, characterization of the gut microbiota in RAU and identification of microbial therapeutic targets are highly warranted. In this study, it was found for the first time that there were concurrent gut dysbacteriosis, microbial dysfunction and immune imbalance in RAU. Although there were no significant differences in the relative abundances of enteroviruses and fungi between RAU patients and healthy individuals, we found that an array of probiotics were depleted while a large number of pathogens were enriched in the gut microbiota of RAU patients. Overall, there is an imbalance between probiotics and pathogenic bacteria. Notably, some *Lactobacillus*, *Bifidobacterium*, *Streptococcus*, and *Enterococcus* species, such as *Lactobacillus ruminis Bifidobacterium animalis*, *S. thermophilus* and *Enterococcus faecium*, have been used as commercial probiotic products (38).

Existing studies suggest that microbiota-driven variations in the inflammatory response regulate the host response to infection (37). A possible explanation is that the imbalance between anti-inflammatory and proinflammatory responses suppresses protective Th17 inflammation and impairs defense against pathogens. In particular, a considerable decrease in the abundance of probiotics, including *A*. *intestine*, and an increase in the abundance of numerous pathogens, including *B*. *fragilis* and *E*. *coli*, were noted in this study. Moreover, pathogen-associated pathways were significantly increased, such as the TCA cycle and tryptophan biosynthesis pathways.

Thalidomide treatment contributes to an increase in the abundance of some probiotics, such as *A. intestine*, and a decrease in the abundance of some pathogens, such as *B. fragilis*. This regulation can be maintained long-term, but thalidomide has no regulatory role for some pathogenic bacteria, suggesting that thalidomide may indirectly alter the gut microbiota by regulating immune factor levels of the host. Probiotics and prebiotics can promote the balance of the intestinal microbiota by regulating specific microbes, and the effects of a suitable combination of synbiotics are beneficial (39). Probiotics, prebiotics, synbiotics and other emerging treatments may be beneficial supplements. Our study indicated that gut dysbacteriosis is a prominent feature of this disease model, and the alleviation and aggravation of gut dysbacteriosis is also consistent with recurrence features. Based on the treatment model and recurrence model, our results demonstrated that thalidomide may differentially regulate gut probiotics and pathogens in RAU according to long-term or short-term patterns. In the long-term pattern, thalidomide is considered to have a persistent effect on the results, that is, at T3, it can still continue the trend of T2; otherwise, it is considered to be a short-term pattern.

**Figure 10:**
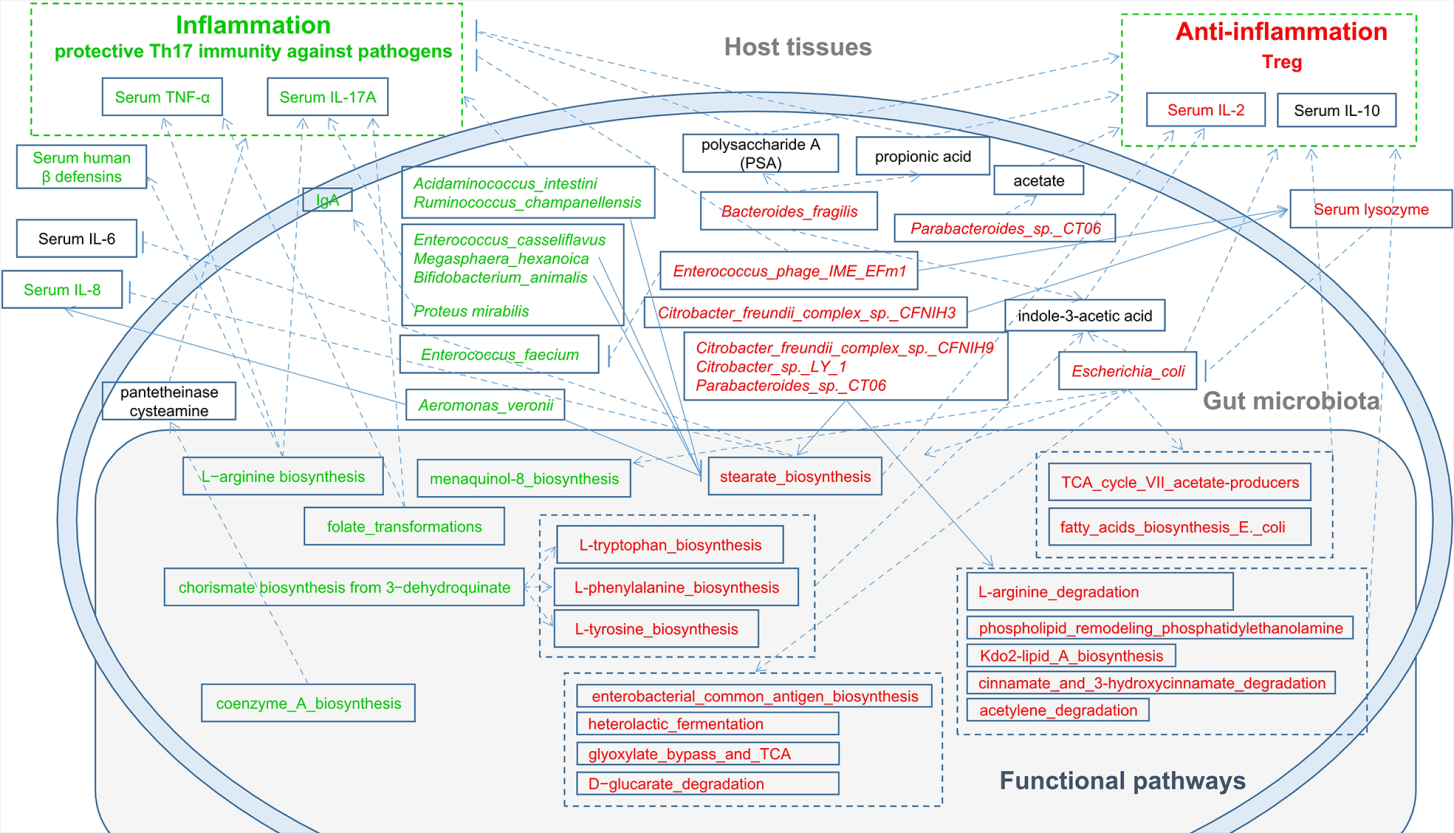
A schematic diagram showing the main functions and cytokines of the gut microbes that had a predicted RAU association. Red text denotes enriched functions, species and cytokines in RAU patients. Green text denotes depleted functions, species and cytokines in RAU patients.

Tryptophan can be used as a biomarker to reflect the occurrence and development of diseases and can also be used to monitor the response to treatment. Tryptophan and other amino acid metabolic pathways are activated in cancers and other diseases (27). Our results indicated that these pathways were significantly enriched in RAU, suggesting an anti-inflammatory role of these pathways in RAU. Based on our results and the literature (40), the possible mechanisms are shown in **Figure 10**. Studies (41, 42) suggest that intestinal dysbacteriosis may promote the pathogenesis of BD through various mechanisms, such as damaging the intestinal mucosal barrier, inducing the overactivation of Th1 and Th17 responses, and reducing the number of Tregs. In addition to oral ulcers, BD patients have systemic manifestations different from those of RAU. Moreover, except for oral lesions, systemic inflammation is absent in RAU. Our study further demonstrates from the microecological and immunological perspectives that BD and RAU are dissimilar and may have different pathogeneses.

The limitations of our study should be considered. Although in this study we investigated changes in species abundance at the metagenomic level, microbial function of the gut microbiota, and serum and saliva levels of immune factors in RAU, animal experiments are absent because there is currently a void regarding animal models exactly simulating RAU in humans. Second, we performed a metagenomic study of saliva microbial samples (data not shown), but enough high-quality metagenomic data were not available due to host exfoliated cell DNA. Therefore, technological breakthroughs in oral and salivary metagenomics are urgently needed to fulfill disease-associated metagenomic profiling.

## Conclusion

In the present study, species-pathway-factor correlation networks facilitated improved metagenomic analysis and helped pinpoint disease- and host-associated shifts in the microbiome’s functional capacity. We observed dynamic shifts in the species composition of the gut microbiota, functional pathways of signature bacteria and immune factor levels in RAU. It was noted that protective Th17 inflammation against pathogens was impaired, contributing to an immunosuppressive microenvironment and probiotic-pathogen dysbiosis. Thalidomide ameliorated gut dysbacteriosis, regulated immune imbalance, and alleviated RAU severity. Probiotics, prebiotics, synbiotics, postbiotics, and other emerging treatments may be beneficial supplements for RAU treatment. With the continuous in-depth study of RAU-related pathogenesis, it may be possible to apply precise treatment of RAU in the future. In conclusion, our findings extend our insights into the metagenome of the gut microbiota and the host in RAU and the regulatory roles of thalidomide, pointing to possible future modalities for RAU prophylaxis and treatment.

## Methods

### Study subjects

This study was approved by the Ethics Committee, Nanjing Stomatological Hospital, Medical School of Nanjing University [2014NL-002(KS)]. The samples and clinical information used in this study were obtained under conditions of informed consent. The diagnostic criteria of RAU patients referred to the criteria previously documented in the literature (43). According to recurrence period and frequency (44, 45), RAU is classified as refractory type (attack at least once per month, totally more than 50% of the time with aphthous ulcers) and common type (attack once 2 or more months) based on the literature and our previous study. According to the severity, RAU is classified as minor, major and herpetiform types classically. Only the patients affected with refractory and minor RAU were included in this study. Individuals with inflammatory diseases, including BD and Crohn’s disease; systematic diseases, including cardiovascular diseases, diabetes and anemia; infectious diseases; and a history of drug abuse were excluded from this study. In this study, 81 RAU patients receiving no medication consisting of antibiotics, corticosteroids or analgesics for at least 1 month along with 44 sex-, age-, and BMI-matched healthy controls were recruited according to the previous studies on gut microbiota and metagenomic analysis (46–48). Information on lifestyle, oral health status, clinical characteristics of ulcers, and blood test-related data were obtained. The participants in this study were healthy controls labeled Control and RAU patients at three points labeled T1, T2, and T3 [T1: before thalidomide administration (thalidomide 50mg nightly) (7, 49); T2: one month after thalidomide administration; T3: one month after thalidomide withdrawal].

### Sample collection and processing

Serum, saliva and fecal samples were collected from participants at approximately 8 am before breakfast. Venous blood from each individual was collected to harvest serum samples. Fecal samples were collected using an OMNIgene·GUT stool/feces sampling kit (DNA Genotek, Ottawa, Canada). Unstimulated whole saliva was collected using a sampling tube and an ORAgene·DNA saliva sampling kit (DNA Genotek, Ottawa, Canada). All samples were immediately frozen and stored at −80°C until analysis.

### Biochemical assays

Serum and saliva samples for biochemical assays were analyzed by ELISA. IL-2, IL-4, IL-6, IL-8, IL-10, IL-17A, IL-23, TNF-α, IFN-γ, IgA, IgG, IgM, and IgE were analyzed using ELISA kits (MultiScience, Hangzhou, Zhejiang, China). LL-37, hBD-1, hBD-2, and hBD-3 were detected using ELISA kits (Cusabio, Houston, TX, USA). Lysozyme, complement-3, and complement-4 were measured using ELISA kits (Abcam, Cambridge, MA, USA). ELISA was performed according to the manufacturers’ instructions.

### Metagenomic sequencing

Paired-end metagenomic sequencing was performed on the Illumina HiSeq 4000 platform (BGI-genomics, China) with an insert size of 350 bp and paired-end (PE) reads of 150 bp for each sample. After removing adaptors and low quality (quality ≤ 20) and ambiguous bases from the raw reads, the remaining reads were aligned to human genome reference (hg19) by SOAPaligner (v2.22, parameters: -m 280 -x 420 -r 1 -l 32 -s 75 -c 0.9) to remove human host DNA contamination.

### Microbiome characterization

All metagenomic sequencing data were processed using the same extensive processing pipeline: bacterial, archaeal, viral, and microeukaryote abundances were determined using Kraken2 (50) and corrected by Bracken (51). A cladogram was produced by GraPhlAn (52). Microbial pathways and abundances were determined using HUMAnN3 (53)(nucleotide-database: chocophlan; protein-database: uniref90) software.

### Statistical analyses

To compare the collected phenotypes of the disease cohort with the population controls, categorical data were tested using edgeR (54) (calcNormFactors: trimmed mean of M-values method). The statistical analysis for differentially expressed (DE) was done using edgeR (glmLRT test). Pathways or species with FDR ≤ 0.05 were set as cutoff values to be considered differentially expressed. ANOVA (Tukey-HSD) test was used to assess differences in taxonomic and functional diversity.

To test for differentially distributed pathways across genotypes, data obtained with HUMAnN3 (pathway composition) were fitted into limma’s model (55) using subjects as blocking variables. Since both software programs quantify biological units using relative counts (HUMAnN2 uses “copies per million”), we transformed these data into logarithmic values using the formula log2(x + 0.1), where x is the relative counts. The obtained *P* values were corrected using the Benjamini–Hochberg correction method.

The Spearman coefficient was used to evaluate the correlation between phenotypes and the correlation between microbiome features. Correlations with corresponding empirical *P* values less than 0.05 were retained. Correlation coefficients with magnitudes of 0.3 or greater were selected for visualization in Cytoscape.

The AUC value of one hundred ninety statistically significantly differentially abundant species (edgeR-TMM, FDR ≤ 0.05) between CONTROL vs. T1 calculated by the ROCR package, and thirty-eight species with AUC>0.7 were discriminated cases (T1 phenotype) from controls with an AUC value according to the adabag package (56) (boosting, mfinal=100). All relevant pathways, factors, and species were screened based on Spearman’s correlation coefficient. Correlation coefficients with magnitudes of 0.2 or greater were selected for visualization in Cytoscape (v. 3.8.2). For model training, the adabag (R package) was used to create an adaboost model. Half of the samples were randomly selected as the training set and the other half as the test set. About the parameter set of training, we defined 1000 trees for fitting, and set parameter shrinkage = 0.01 and cv. folds = 5.

## Data availability statement

The datasets presented in this study can be found in online repositories. Sequences generated from this study are deposited in the China National GeneBank (CNGB) at https://db.cngb.org/cnsa/ (accession number: CNP0001744).

## Ethics statement

The studies involving human participants were reviewed and approved by Nanjing Stomatological Hospital, Medical School of Nanjing University [2014NL-002(KS)]. The patients/participants provided their written informed consent to participate in this study.

## Author contributions

XW, KX, FH, JH, QL, and WW had full access to all data and take responsibility for the integrity of the data and accuracy of data analysis. The study concept and design were provided by XW, QH, FY and WW. Acquisition, analysis or interpretation of data was carried out by all authors. Drafting of the manuscript was performed by QL and FH. All authors critically revised the manuscript for important intellectual content. Statistical analysis was carried out by KX and JH. Funding was obtained by XW, QH, FY, WW. Administrative, technical or material support was provided by XW, JH and WW. The study was supervised by QH, FY and WW. All authors contributed to the article and approved the submitted version.

## Funding

This work was supported by the National Natural Scientific Foundation of China (81870767 & 81570978), the Key Project of Science and Technology Department of Jiangsu Province (BL2014018), the Project of Jiangsu Provincial Medical Youth Talent (QNRC2016118), and the Nanjing Clinical Research Center for Oral Diseases (2019060009).

## Acknowledgments

We would thank BGI-genomics (Shenzhen, China) for technical assistance and bioinformatics analysis. 

## Conflict of interest

The authors declare that the research was conducted in the absence of any commercial or financial relationships that could be construed as a potential conflict of interest.

## Publisher’s note

All claims expressed in this article are solely those of the authors and do not necessarily represent those of their affiliated organizations, or those of the publisher, the editors and the reviewers. Any product that may be evaluated in this article, or claim that may be made by its manufacturer, is not guaranteed or endorsed by the publisher.
