# Supplementary figures and images for: A metagenome-wide association study of the gut microbiota in recurrent aphthous ulcer and regulation by thalidomide
Source: Front Immunol. 2022 Oct 19;13:1018567. doi: 10.3389/fimmu.2022.1018567 (PMC9626999; doi:10.3389/fimmu.2022.1018567)

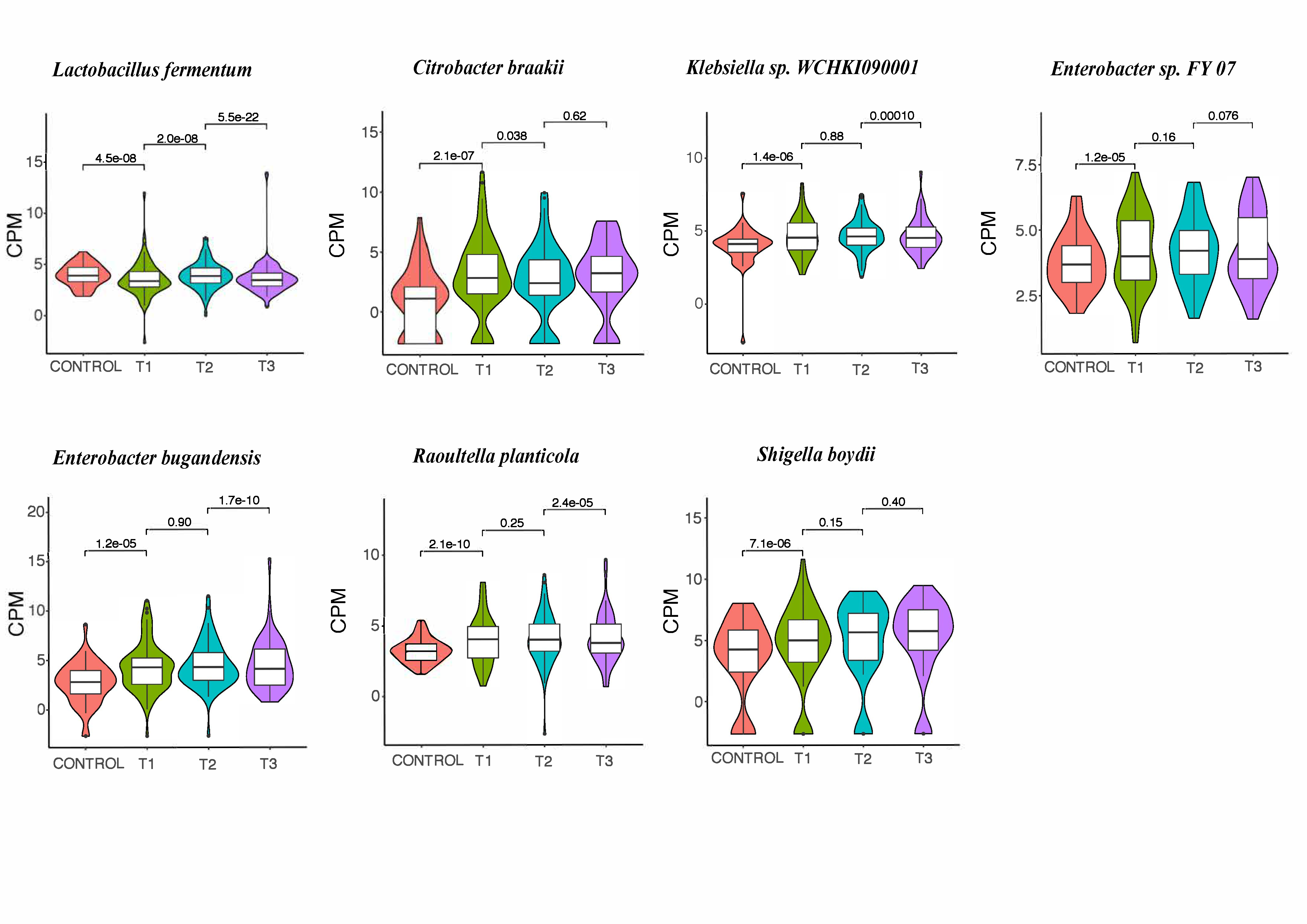

Supplement: Supplementary file 1 [file Image_1.tiff]

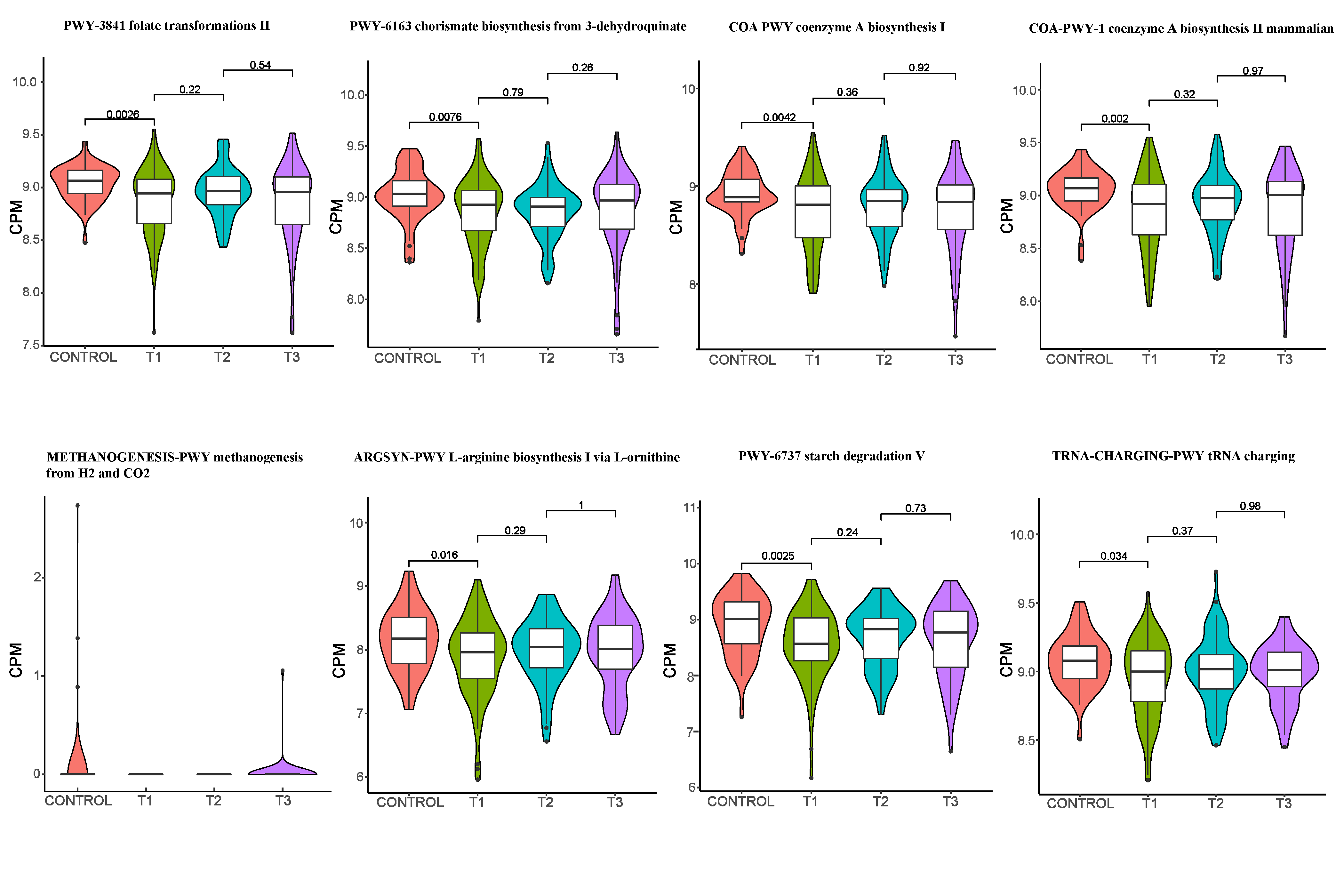

Supplement: Supplementary file 2 [file Image_2.tiff]

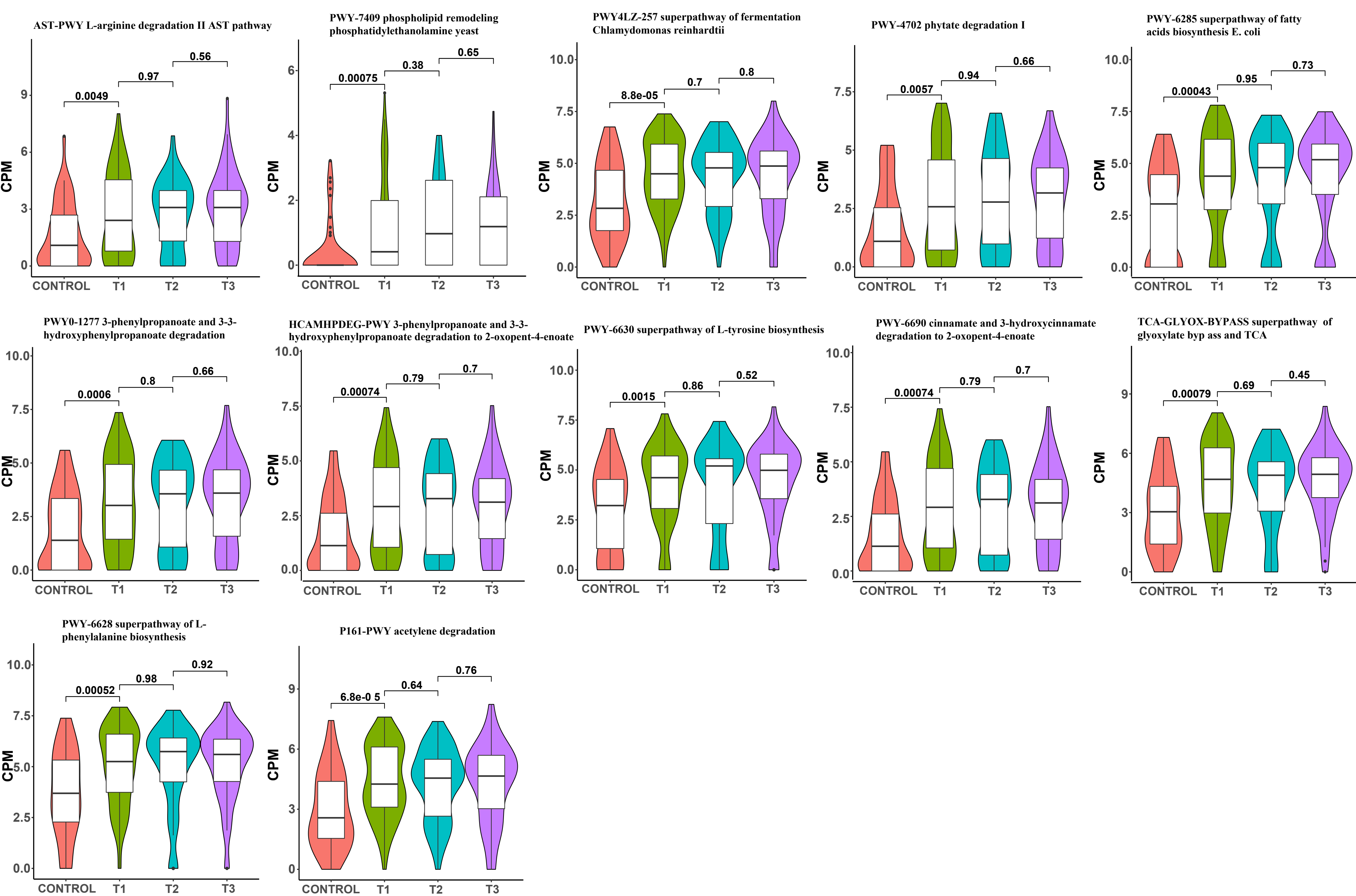

Supplement: Supplementary file 3 [file Image_3.pdf]

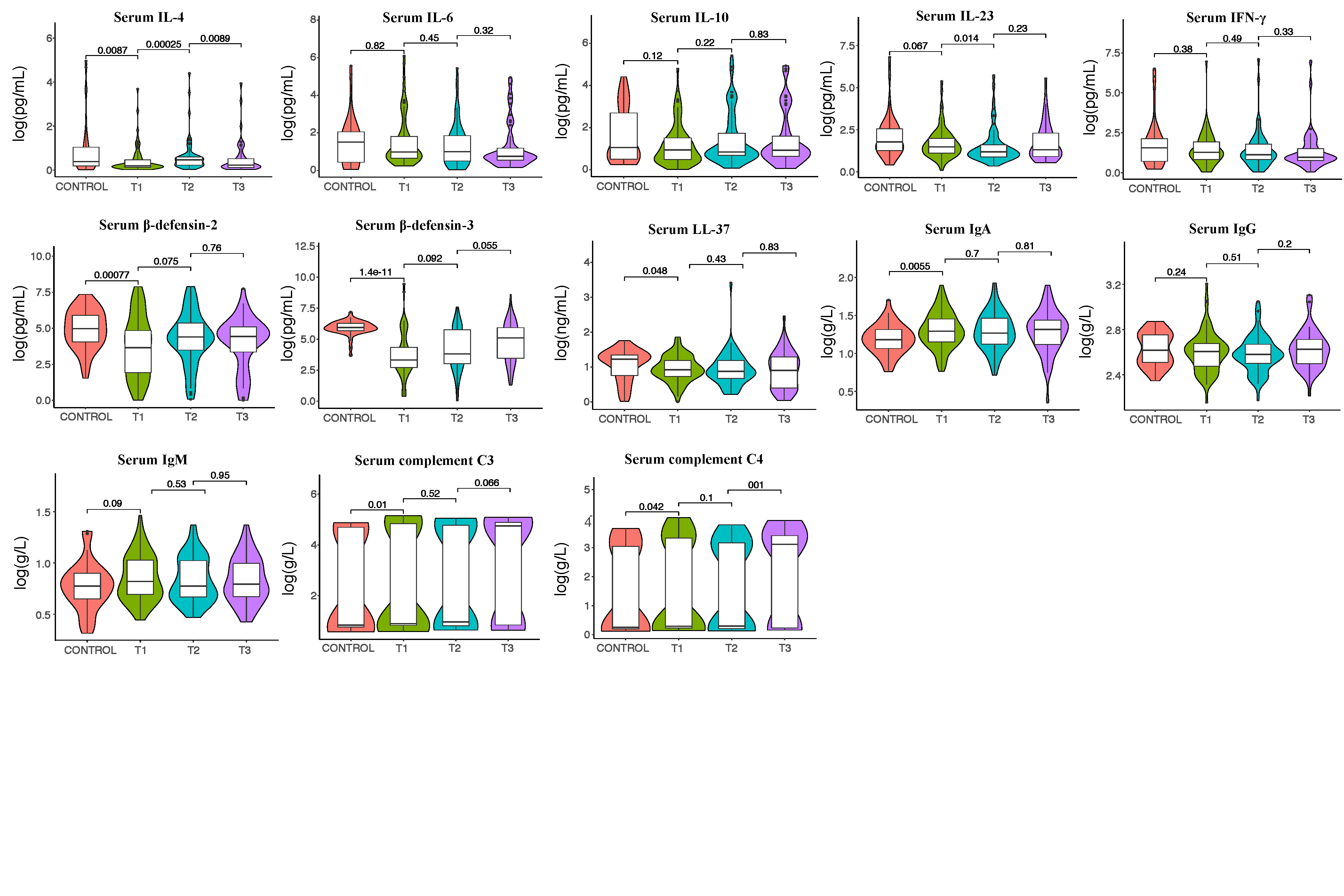

Supplement: Supplementary file 4 [file Image_4.tiff]

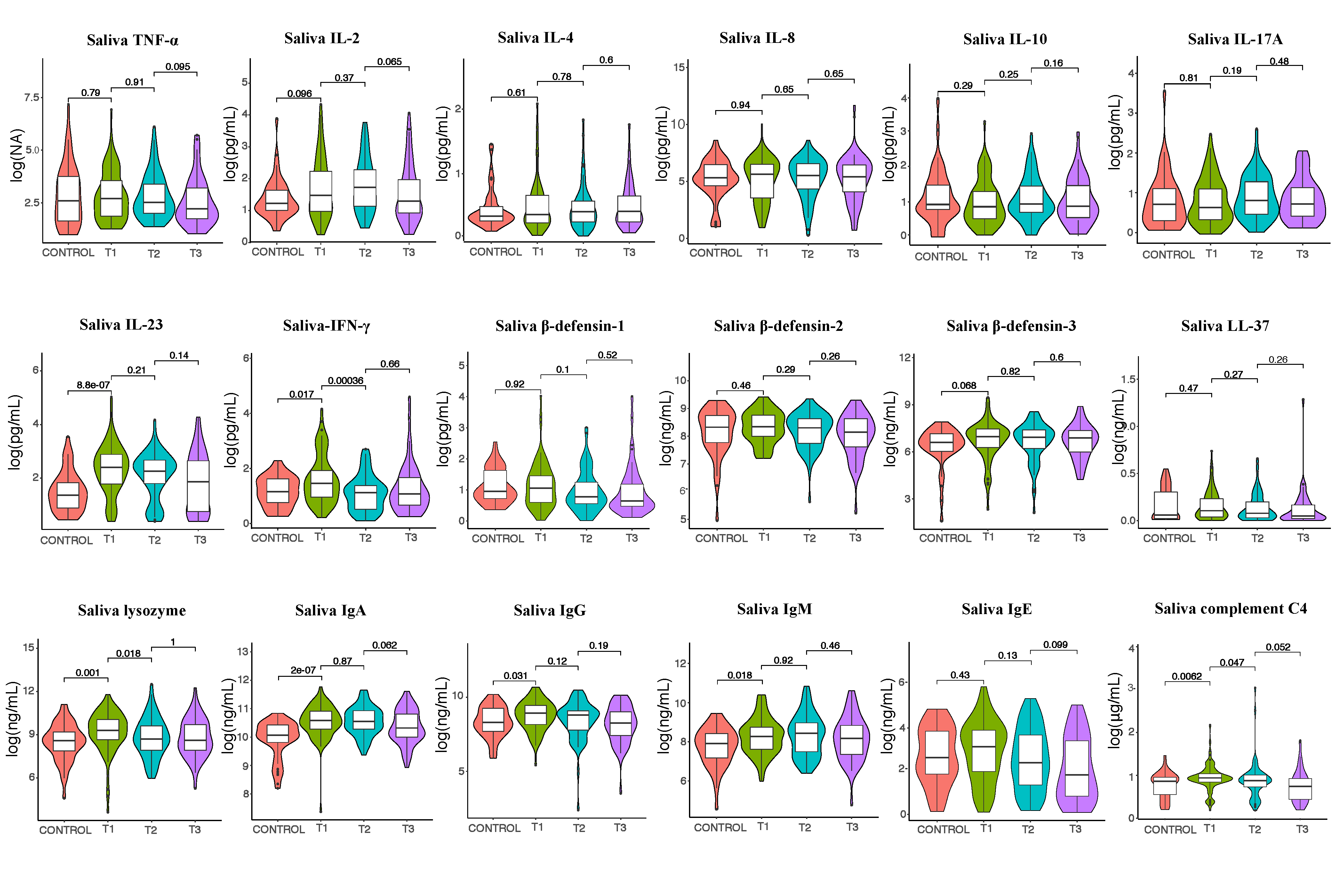

Supplement: Supplementary file 5 [file Image_5.tiff]
