# Supplementary material for: A metagenome-wide association study of the gut microbiota in recurrent aphthous ulcer and regulation by thalidomide
Source: Front Immunol. 2022 Oct 19;13:1018567. doi: 10.3389/fimmu.2022.1018567 (PMC9626999; doi:10.3389/fimmu.2022.1018567)
Supplement: Supplementary file 6 [file Table_1.docx]

**Supplementary Table 1** Baseline information of the Control and RAU patient groups

| **Characteristic** | **Group** | | ***t* /*χ^2^*** | ***P* value** |
| --- | --- | --- | --- | --- |
|  | **Control** | **RAU patient** |  |  |
|  | (n=44) | (n=81) |  |  |
| **Age**, y | 46.99±10.01 | 44.98±9.30 | -1.123 | 0.264 |
| **Gender**, No. (%) |  |  | 0.021 | 0.887 |
| Female | 22(50.00) | 38(46.91) |  |  |
| Male | 22(50.00) | 43(53.09) |  |  |
